# McConnell’s sign unveiled

**DOI:** 10.1186/1532-429X-14-S1-P88

**Published:** 2012-02-01

**Authors:** Valentyna Ivanova, Mark Doyle, June Yamrozik, Ronald B Williams, Geetha Rayarao, Moneal Shah, Tariq Cheema, Robert W Biederman

**Affiliations:** 1 Allegheny General Hospital, Pittsburgh, PA, USA

## Summary

McConnell's sign is well described in echocardiography as a harbinger of severe RV failure and more extensive arterial thrombus burden but its mechanical basis has never been satifactorily defined despite multiple current theories. CMR RF tissue-tagging now unveils the mechanism.

## Background

Evidence of echocardiographic RV ‘strain’ in the setting of a large, clinically important, often devasting pulmonary embolism (PE) is defined by the presence of paradoxical, often hyperdynamic RV apical contraction in the setting of severely dysfunctional RV function; so denoted ‘McConnell’s sign’. Despite its original observation by its namesake in 1996, it has neither been observed via CMR nor more importantly, while many theories exist, mechanistically explained.

### Hypothesis

We hypothesize that McConnell’s sign (MS) is observable utilizing CMR and can be explained via CMR deformational analysis.

## Methods

Modestly stable patients who had CT confirmed large (>one pulmonary branch obstruction and/or saddle PE) were evaluated via echocardiography and CMR. CMR radio-frequency tissue tagging was performed to determine midwall intramyocardial deformation patterns with superimposition of quiver plot technique to define direction and amplitude of myocardial displacements assuming homogenous tissue strain (%S).

## Results

Eleven patients (3 male; 59±9 years) were identified from CT of which 7 had echocardiograms performed of which 5 were MS+. Four patients had body habitus permitting CMR examination (GE Excite1.5T, Milwaukee, WI)within 36-96 hours of echocardiogram. The LV was hyperdynamic,small and under-filled in all (LVEF=72-81%; LVEDV <2SD below LLN). As expected, the RV was severely dysfunctional and dilated in all (RVEF=23-32%; RVEDV>2SD above ULN). MS+ was present in 2 patients but absent in 2 patients (imaged >72 hours after CT/echo). All 4 patients underwent RF tagging in multiple projections with %S and were compared to historic controls. Circumferential (%S_C_), radial (%S_R_) and meridional(%S_L_) were interrogated. In MS+, septal%S_L_ was markedly augmented (87%) as compared to MS- (42%) and historic controls (15-18%) and less than the moderately augmented lateral wall (58%) in MS+. RV apical %S_L_ was negligible(<1%). A distinct base-apex gradient in %S_L_ was present,highest at the LV apex adjacent tethered RV insertion point fibers (p<0.05 for all vs. historic controls). %S_C_ and %S_R_, while augmented, did not demonstrate a directional component consistent with observed MS+.

## Conclusions

Standard CMR permits detection of McConnell’s sign in patients with large, relevant pulmonary embolisms. CMR appears for the first time to sufficiently explain the mechanism behind the long-observed mechanism McConnell’s sign: in the setting of substantial preload reduction and reflex hyperadrenergic up-regulation, RF tissue tagging demonstrates markedly augmented LV septal intramyocardial deformations, such that tethered RV insertion point fibers are acted upon via the hyperdynamic LV apex permitting translation with pronounced but paradoxical RV apical passive deformation.

## Funding

Internal.

